# ﻿Two new species of *Platensina* Enderlein (Diptera, Tephritidae, Tephritinae, Dithrycini) from India

**DOI:** 10.3897/zookeys.1092.80645

**Published:** 2022-04-06

**Authors:** K. J. David, D. L. Hancock, K. Sachin, R. G. Gracy, S. Salini

**Affiliations:** 1 National Bureau of Agricultural Insect Resources, Bengaluru-560024, Karnataka, India National Bureau of Agricultural Insect Resources Bangalore India; 2 60 South Street, Carlisle, Cumbria CA1 2EP, UK unaffiliated Cumbria United Kingdom

**Keywords:** Identification key, *Ludwigia*, Meghalaya, Onagraceae, Platensinina, south India

## Abstract

Two new species of *Platensina* Enderlein, *P.rabbanii* David & Hancock, **sp. nov.**, and *P.flavistigma* David & Hancock, **sp. nov.**, are described from Meghalaya and southern India, respectively. *Platensinarabbanii* can be differentiated from *P.alboapicalis* Hering by the presence of a single hyaline indentation in cell r_1_ and the apical hyaline band in cell r_2+3_ restricted to the apex; *P.flavistigma* differs from *P.quadrula* Hardy by the presence of a yellow/fulvous pterostigma and shape of the epandrium. DNA barcode sequences of *P.acrostacta* (Wiedemann), *P.flavistigma* and *P.platyptera* Hendel were obtained and reported. Postabdominal descriptions and illustrations of *P.acrostacta*, *P.platyptera* and *P.zodiacalis* (Bezzi) are also provided along with keys to all 23 species and the 7 known from India.

## ﻿Introduction

*Platensina* Enderlein is predominantly an Oriental and Australasian genus with 24 species recognized by Norrbom et al. (1999) [some generically misplaced] and 21 by Hancock (2012). They are medium-sized flies with broad, dark brown wings with hyaline indentations and spots. Host plants are not recorded except for *Platensinaacrostacta* (Wiedemann), reared from stem galls of an undetermined *Ludwigia* species in southern India (Hardy 1973; Hancock 2012). Agarwal and Sueyoshi (2005) listed five species from India, while David and Ramani (2011) provided keys to four species from peninsular India and the Andaman and Nicobar Islands. Hancock (2012) recorded *P.platyptera* Hendel and *P.quadrula* Hardy from India, regarded records of *P.amplipennis* (Walker) from India as misidentifications of *P.platyptera* and provided a key to species of *Platensina*. In this paper two new species, one collected from Meghalaya and one from southern India, are described, along with descriptions of postabdominal structures of other species recorded from India except *P.tetrica* Hering and *P.fulvifacies* Hering, as specimens of these two taxa were not available for study. A key to species of *Platensina* recorded from India is provided, together with a revised key to all known species.

## ﻿Material and methods

Specimens studied are deposited in the National Insect Museum, ICAR – National Bureau of Agricultural Insect Resources, Bengaluru, India (**NIM**).

Collections were done by sweep netting. Images of specimens were taken using a Leica DFC 420 camera mounted on a Leica M205A stereo zoom microscope; images of genitalia were taken using an 8 MP camera temporarily attached to a Leica DM 1000 compound research microscope; the images were stacked and combined to a single image using Combine ZP (Hadley 2011). Measurements of male and female genitalia were taken using Leica Automontage Software, LAS 3.4. Terminology adopted here follows White et al. (1999) and wing terminology by Cumming and Wood (2017).

One hind leg was removed from one specimen of each of three species and used for DNA extraction. The DNA extraction was performed using a DNeasy Blood and Tissue Kit (Qiagen India Pvt. Ltd.) following the manufacturers’ instruction. For the molecular study, the standard DNA barcoding region of the mitochondrial COI gene was sequenced and the PCR was performed using the Universal COI primers (LCO1490/HCO2198) (David et al. 2020). The sequences were annotated using NCBI Blast tools and submitted to the NCBI GenBank Database where accession numbers were obtained (*Platensinaflavistigma* – MT019893; *Platensinaacrostacta* – MT019891; *Platensinaplatyptera* – MW448367).

The pairwise genetic distance between three species of *Platensina* viz., *P.acrostacta*, *P.platyptera* and *P.flavistigma* has been calculated using mitochondrial COI gene sequences. Analyses were conducted using the Maximum Composite Likelihood model (Tamura et al. 2004). This analysis involved 4 nucleotide sequences. Codon positions included were 1^st^+2^nd^+3^rd^. All positions with less than 95% site coverage were eliminated, i.e., fewer than 5% alignment gaps, missing data, and ambiguous bases were allowed at any position (partial deletion option). There were a total of 557 positions in the final dataset. Evolutionary analyses were conducted in MEGA11 (Tamura et al. 2021).

## ﻿Results

### ﻿Taxonomy

#### ﻿ Platensina

Taxon classificationAnimaliaDipteraTephritidae

Enderlein, 1911

CD4538D0-ABB2-50A8-8FFD-67BE8F501E55

Platensina Enderlein, 1911: 454. Type species: Platensinasumbana Enderlein.
Tephrostola Bezzi, 1913: 153. Type species: Trypetaacrostacta Wiedemann.

##### Diagnosis.

Medium-sized flies (4–5 mm long), with frons as wide as long, three frontal setae, two orbital setae, well developed ocellar setae. First flagellomere shorter than face, with short-pilose arista, face usually fulvous, black in males of a few species. Scutum grey pubescent with yellow-white reclinate setulae; scutellum flat with one or two pairs of setae, apical pair less than half length of basal setae or absent. Wing broad, often distinctly angled along posterior margin, dark brown with hyaline indentations and subhyaline spots. Abdomen predominantly black with fulvous lateral regions. Epandrium broad, without demarcation between epandrium and lateral surstylus, lateral surstylus broad, epandrium elongate-oval in posterior view; medial surstylus with well sclerotised prensisetae (lateral one broader than medial one), proctiger not higher than epandrium, glans of phallus stout, with single sclerotised acrophallus. Taeniae short (0.25 of eversible membrane); spicules on eversible membrane conical; aculeus dorsoventrally flattened, tip conical, with reduced preapical setae; spermathecae club-shaped, with numerous papillae.

### ﻿Key to species of *Platensina* from India

**Table:** 

1	Apex of wing hyaline from middle of cell r_2+3_ to cell cua (Fig. 6); posterior wing margin not distinctly angled near apex of cell cua	***P.rabbanii* David & Hancock sp. nov.**
–	Wing with a hyaline spot restricted to apex of cell r_4+5_ (e.g. Figs 16, 20, 21); posterior wing margin distinctly angled near apex of cell cua	**2**
2	Wing with discal spots small and often indistinct or subhyaline; cell r_1_ with 0–2 small hyaline indentations from costa in basal portion beyond stigma, often not crossing cell; cell cua with 3 small, isolated hyaline marginal spots and with or without additional small, isolated discal spots; holotype illustrated by Hering 1939a, fig. 14	***P.tetrica* Hering, 1939**
–	Wing with distinct hyaline discal spots; hyaline indentations in basal portion of cell r_1_ with at least the basal one broad and crossing into cell r_2+3_; cell cua with 2–3 hyaline marginal indentations, the basal pair usually elongate but often divided medially into 2 separate spots	**3**
3	Wing (Figs 20, 21) with pterostigma entirely dark brown to black, 2 elongate marginal hyaline indentations in cell r_1_, both crossing vein R_2+3_ into cell r_2+3_, no marginal preapical hyaline spots in cell r_2+3_, large hyaline spots near base of cell r_4+5_ and near base and apex of cell dm, 1 marginal spot in cell m near apex of vein CuA, 2 indentations in cell cua and 1 or 2 spots along margin of anal lobe	**4**
–	Wing markings not as above (Figs 16, 31, 32, 42, 43); pterostigma usually with a subhyaline or fulvous basal patch or spot, marginal preapical hyaline spot in cell r_2+3_ usually present, and cell m usually with 2 or 3 hyaline marginal spots	**5**
4	Face black in male, yellow in female; wing (Figs 20, 21) with basal spot in cell dm not distinctly larger than apical spot and not crossing or almost crossing cell; hyaline indentations in cell cua of approximately equal length, almost crossing cell but basal spot sometimes narrowly divided medially	***P.acrostacta* (Wiedemann, 1824)**
–	Face yellow in both sexes; wing with basal spot in cell dm distinctly larger than apical spot and crossing or almost crossing cell; basal hyaline indentation in cell cua much smaller than second indentation or broadly divided medially into 2 small spots; holotype illustrated by Hering 1941, fig. 4	***P.fulvifacies* Hering, 1941**
5	Wing (Fig. 16) with base and pterostigma largely fulvous to pale brown, contrasting with rest of wing; cell m without an isolated anterobasal hyaline spot	***P.flavistigma* David & Hancock, sp. nov.**
–	Wing (Figs 31, 32, 42, 43) with pattern variable but uniformly dark brown with hyaline spots and markings; cell m with an isolated anterobasal hyaline spot	**6**
6	Scutellum with apical and basal setae	***P.platyptera* Hendel, 1915**
–	Scutellum with only basal setae	***P.zodiacalis* (Bezzi, 1913)**

### ﻿Key to all known species of *Platensina*

Modified from Hancock (2012).

**Table:** 

1	Wing broad and almost circular beyond basal third, apex evenly rounded and entirely dark, without hyaline discal or marginal spots or indentations except for pair of small costal spots at bases of pterostigma and cell r_1_ adjacent to apices of veins Sc and R_1_, respectively; illustrated by Hardy 1974, fig. 129 [Philippines (Luzon)]	***P.bezzii* Hardy, 1974**
–	Wing often broad but distinctly longer than wide, apex at least slightly produced and with at least a hyaline apical spot in cell r_4+5_; usually with hyaline discal and marginal spots or indentations	**2**
2	Wing (Fig. 6) with hyaline apical band distinctly crossing veins R_4+5_ and M_1_ into cells r_2+3_ and m_1_	**3**
–	Wing (Figs 10, 16, 20, 21, 32, 43) with oval or quadrate hyaline apical spot confined to cell r_4+5_	**7**
3	Male wing without hyaline spots or indentations apart from small marginal indentation in cell r_1_ at apex of vein R_1_ and crescentic hyaline apex; female wing with crescentic hyaline apex plus hyaline marginal spots and indentations and subbasal hyaline spot in cell dm but no spot near base of cell r_4+5_; illustrated by Wang 1998, fig. 253–4 [China (Yunnan)]	***P.nigripennis* Wang, 1998**
–	Male wing (where known) with one broad or 2 narrow marginal hyaline indentations in cell r_1_ near apex of vein R_1_ and often a spot near base of cell r_4+5_; female wing (where known) with hyaline medial spot close to line of crossvein r-m and often a spot near base of cell r_4+5_	**4**
4	Wing of both sexes without hyaline spot near base of cell r_4+5_ and hyaline apex in cell m separate from the 2 hyaline marginal spots; hyaline marginal indentation in cell r_1_ broad and rectangular in male, divided into elongate indentation and 2 small round spots in female; male illustrated by Hardy and Drew 1996, fig. 167 [Australia (Queensland)]	***P.parvipuncta* Malloch, 1939**
–	Wing of both sexes (where known) with hyaline spot near base of cell r_4+5_; apex with hyaline band in cell m enclosing one or both marginal spots, leaving no more than a single separate marginal spot; hyaline indentation from costa in cell r_1_ not as above	**5**
5	Male unknown; female posterior to vein R_4+5_ with spots in cells r_4+5_ (1, at base), bm (1, near apex), dm (1, near middle), m (1 marginal near apex of vein CuA), cua (3, 2 basal and 1 near middle) and anal lobe (1); not illustrated [Taiwan]	***P.apicalis* Hendel, 1915**
–	Female unknown; male with 1 or 2 hyaline indentations in cell r_1_ near apex of vein R_1_ and 1 or no hyaline marginal spots in cell m near apex of vein CuA separate from the apical hyaline area, large hyaline spots at base of cell r_1_ and at basal third of cell dm, and 2 elongate marginal indentations in cell cua [India and Burma]	**6**
6	Wing in cell r_1_ with 2 hyaline indentations near apex of vein R_1_; cell m with one hyaline marginal spot in addition to apical hyaline area; cell cua with hyaline marginal indentations reaching or almost reaching vein CuA; illustrated by Hering 1938, fig. 50 [NE Burma]	***P.alboapicalis* Hering, 1938**
–	Wing (Fig. 6) with cell r_1_ with 1 hyaline indentation near apex of vein R_1_ and cell m without a hyaline marginal spot in addition to apical hyaline area; cell cua with hyaline marginal indentations ending at or before middle of cell	***P.rabbanii* David & Hancock, sp. nov.**
7	Wing with cell c and basal two-thirds of pterostigma hyaline; hyaline marginal spots (including 2 in cell m_1_ and 3 in cell cua) present but pale discal spots absent; head with 1–2 pairs of frontal setae; illustrated by Hardy 1974, fig. 127 [Philippines (Luzon)]	***P.amita* Hardy, 1974**
–	Wing with cell c not entirely hyaline and pterostigma with at most a hyaline basal spot; both hyaline marginal and pale discal spots usually present; head with 3 pairs of frontal setae	**8**
8	One pair of scutellar setae, apicals absent; illustrated by Bezzi 1913, fig. 65, Hardy 1973, pl. v, fig. 45, Hardy 1974, fig. 130, Wang 1998, fig. 256 and this paper, figs 42–43 [India and Sri Lanka to Philippines, Indonesia and northern Australia]	***P.zodiacalis* (Bezzi, 1913)**
–	Two pairs of scutellar setae, apicals present	**9**
9	Wing cell r_2+3_ with 2 narrow marginal hyaline spots or indentations; marginal hyaline indentations in cell r_1_ close to apex of vein R_1_ normally reduced in cell r_2+3_ to isolated spots in yellowish field; illustrated by Hancock 2012, figs 3–4 [Indonesia to NE Australia and Solomon Islands]	***P.ampla* de Meijere, 1914**
–	Wing cell r_2+3_ with 0–1 narrow marginal hyaline spots or indentations; marginal hyaline indentations in cell r_1_ close to apex of vein R_1_ present or reduced in cell r_2+3_ but not reduced to isolated spots in a yellowish field	**10**
10	Wing with single large marginal hyaline indentation in cell r_1_ close to apex of vein R_1_ that extends across cell r_2+3_ and is aligned with posterior indentation in cell cua that crosses cell; cell m with single hyaline marginal spot placed close to apex of vein CuA; illustrated by Hardy 1974, fig. 128 [Philippines (Luzon)]	***P.aptata* Hardy, 1974**
–	Wing with 2 marginal hyaline indentations in cell r_1_ close to apex of vein R_1_, the distal one sometimes reduced and largely united with basal one, sometimes both reduced to small marginal spots or single indistinct pale area; cells m and cua not both with a single hyaline marginal spot or band	**11**
11	Wing cell m with 3 hyaline marginal spots or indentations	**12**
–	Wing cell m with at most 2 hyaline marginal spots or indentations	**13**
12	Wing cell dm with 3 rounded hyaline spots; cell m with 2 hyaline spots in anterobasal quarter; illustrated by Hardy and Drew 1996, fig. 168 [Australia (Queensland)]	***P.trimaculata* Hardy & Drew, 1996**
–	Wing cell dm with 2 rounded hyaline spots; cell m with 1 hyaline spot in anterobasal quarter; illustrated by Wang 1998, fig. 251 [Japan (Ryukyu Is), Taiwan and China (Sichuan, Guangxi)]	***P.assimilis* (Shiraki, 1968)**
13	Wing cell r_4+5_ with very small apical hyaline spot; illustrated by Hardy 1988, fig. 22 [Indonesia (Java, Sumba)]	***P.sumbana* Enderlein, 1911**
–	Wing cell r_4+5_ with large apical hyaline spot filling all or most of cell apex	**14**
14	Wing cell r_1_ with 2 large and quadrate hyaline indentations, distal one broader than wide, plus subapical posterior spot not reaching costa; cell m with 2 elongate hyaline indentations, the anterior one much smaller and narrower than the posterior one; cell cua with 3 broad hyaline indentations, the basal pair crossing or almost crossing cell, plus basally with extension of large hyaline mark in anal lobe; male with distinct white or silvery parafacial stripes (females unknown)	**15**
–	Wing markings not as above, distal hyaline indentation in cell r_1_ narrower than wide and/or one or both marks often reduced or absent, subapical spot in cell r_1_, when present, small and marginal at costa or crossing cell, and marginal marks in cell m usually subequal in size; male without white or silvery parafacial stripes	**16**
15	Wing (Figs 9, 10, 16) with dark markings anterior to vein R_4+5_ pale brown to yellow-brown in basal two-thirds and dark brown elsewhere; pterostigma with basal spot yellow; hyaline spot at base of cell r_4+5_ circular; head with parafacial stripes white [southern India (Karnataka, Tamil Nadu)]	***P.flavistigma* David & Hancock, sp. nov.**
–	Wing with dark markings uniformly dark brown; pterostigma with basal spot hyaline; hyaline spot at base of cell r_4+5_ oval; head with parafacial stripes silvery; illustrated by Hardy 1973, fig. 149 [Thailand, Cambodia and Vietnam]	***P.quadrula* Hardy, 1973**
16	Wing cell r_2+3_ without small and narrow marginal spot posterior to apex of vein R_2+3_ and pterostigma without hyaline basal spot; cell m with 1 small semicircular marginal hyaline spot near apex of vein CuA	**17**
–	Wing cell r_2+3_ with small and narrow marginal spot below apex of vein R_2+3_ and pterostigma often with hyaline basal spot; cell m usually with 2 small and often elongate marginal hyaline spots	**18**
17	Face largely black in male, yellow in female; basal spot in cell dm not distinctly larger than apical spot and not crossing or almost crossing cell; hyaline indentations in cell cua of approximately equal length, almost crossing cell but basal spot sometimes narrowly divided medially; illustrated by Bezzi 1913, fig. 57, Hancock 2012, fig. 1 and this paper, figs 20–21 [India and Sri Lanka to southern China (Yunnan) and Cambodia]	***P.acrostacta* (Wiedemann, 1824)**
–	Face yellow in both sexes; basal spot in cell dm distinctly larger than apical spot and crossing or almost crossing cell; basal hyaline indentation in cell cua much smaller than second indentation or broadly divided medially into 2 small spots; illustrated by Hering 1941, fig. 4 [India (Maharashtra, Rajasthan)]	***P.fulvifacies* Hering, 1941**
18	Wing with discal spots often subhyaline or indistinct and pale brown; cell r_1_ often with 0–2 small hyaline indentations or spots in basal portion (especially in males) or with 2 indentations often largely fused; cell cua with 2 or 3 small and isolated hyaline marginal spots and with or without additional small and isolated discal spot	**19**
–	Wing with discal spots normally hyaline and distinct; cell r_1_ with second hyaline indentation distinct and at most weakly joined to first; cell cua with 2–3 hyaline marginal indentations not all small and isolated, basal pair often almost crossing cell or medially divided into separate spots	**20**
19	Wing cell r_1_ with basal hyaline indentations often reduced to 0–2 small hyaline spots (especially in males, better developed in females); cell cua with 3 small and isolated hyaline marginal spots and with or without additional small and isolated discal spots; anal lobe with 2 distinct hyaline marginal spots; illustrated by Hardy 1973, pl. v, fig. 44 and Wang 1998, figs 252 & 255 [India (Tamil Nadu), Thailand and West Malaysia to China (Guangxi, Fujian) and Taiwan]	***P.tetrica* Hering, 1939**
–	Wing cell r_1_ with second hyaline indentation narrow and strap-like or fused with first indentation leaving only small dark costal spot between them; cell cua with 2 small marginal spots; anal lobe with hyaline marginal spots vestigial or absent; illustrated by Bezzi 1913, fig. 64 and Hardy 1973, pl. v, fig. 42 [southern Burma to Vietnam and Indonesia (Sumba)]	***P.euryptera* (Bezzi, 1913)**
20	Wing cell m with a small anterobasal hyaline spot and no marginal spots; cell cua with 2 undivided indentations almost crossing cell; cell r_4+5_ with basal spot large and ovate, much larger than the 2 distinct spots in cell dm; illustrated by Hardy 1973, pl. v, fig. 43 [Thailand, Cambodia and Vietnam]	***P.intacta* Hardy, 1973**
–	Wing cell m with 2 small hyaline marginal spots; cell cua with 2 or 3 indentations, with at least the more distal of the 2 basal indentations divided medially and apical spot small or absent; cell r_4+5_ with basal spot small and circular, not much larger than the 2 distinct spots in cell dm	**21**
21	Wing evenly rounded posteriorly, not distinctly angled basal of end of vein CuA and with numerous distinct discal spots, 1–2 in cell br, 3 in cell r_4+5_, 1–2 in cell r_4+5_, 2 in cell dm and 1 anterobasally in cell m; posterior marginal spot in cell m distinctly larger and broader than anterior marginal spot; illustrated by Wang 1998, fig. 259–260 [Japan (Ryukyu Is), China (Sichuan) and possibly Indonesia (West Papua)]	***P.shirouzui* (Ito, 1984)**
–	Wing distinctly angled posteriorly, broadest just basal of end of vein CuA and with only 3 distinct discal spots, 1 at base of cell r_4+5_ and 2 in cell dm; posterior marginal spot in cell m not distinctly larger and broader than anterior marginal spot	**22**
22	Apical scutellar setae distinct, about half length of basals; anal lobe of wing with hyaline marginal spots vestigial or absent; posterior hyaline marginal spot in cell m narrow, elongate and perpendicular; illustrated by Hancock 2012, fig. 2 [southern Thailand to Indonesia (Java, Sulawesi)]	***P.amplipennis* (Walker, 1860)**
–	Apical scutellar setae weak, about quarter length of basals; anal lobe of wing with hyaline marginal spots round and distinct; posterior hyaline marginal spot in cell m often short and broad; illustrated by Hancock 2012, figs 5–6 and this paper, figs 31–32 [India to Japan (Ryukyu Is), Australia, Solomon Islands and Vanuatu]	***P.platyptera* Hendel, 1915**

### ﻿New species

#### ﻿ Platensina rabbanii

Taxon classificationAnimaliaDipteraTephritidae

David & Hancock sp. nov.

D3B6ED8C-E85E-571D-8A26-650BD863112E

http://zoobank.org/FF51328D-55A7-49B0-934F-48D1D61EF92D

##### Type locality.

India: Meghalaya, East Khasi Hills, Laitsopliah.

##### Type data.

***Holotype*** male, pinned. Original label: “INDIA: Meghalaya, East Khasi Hills, Laitsopliah, 17.iii.2021, Rabbani M. K.” (NIM).

##### Diagnosis.

This species is similar to *P.alboapicalis* Hering from Burma in the presence of an apical hyaline band extending from cell r_2+3_ to cell m_1_ but can be differentiated by the presence of a single hyaline indentation in cell r_2+3_, versus two hyaline indentations and spot in *P.alboapicalis*; the apical hyaline band restricted to the apical one-third of cell r_2+3_, versus the whole of apex of cell r_2+3_ in *P.alboapicalis*; and the hyaline indentations in cell cua ending well before vein CuA, unlike in *P.alboapicalis* where they almost reach vein CuA.

**Figure 1.**
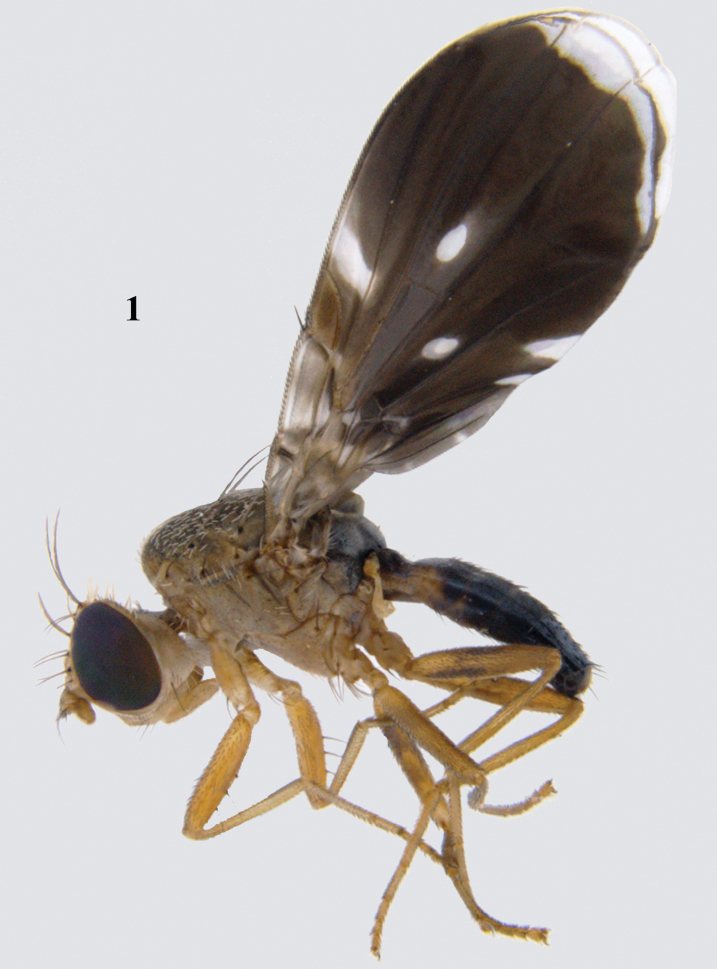
Habitus (lateral) of male of *Platensinarabbanii* David & Hancock, sp. nov.

##### Description.

**Male.** Medium-sized fly (4.03 mm long) with broad, dark brown wing with hyaline apex.

**Figures 2–8.**
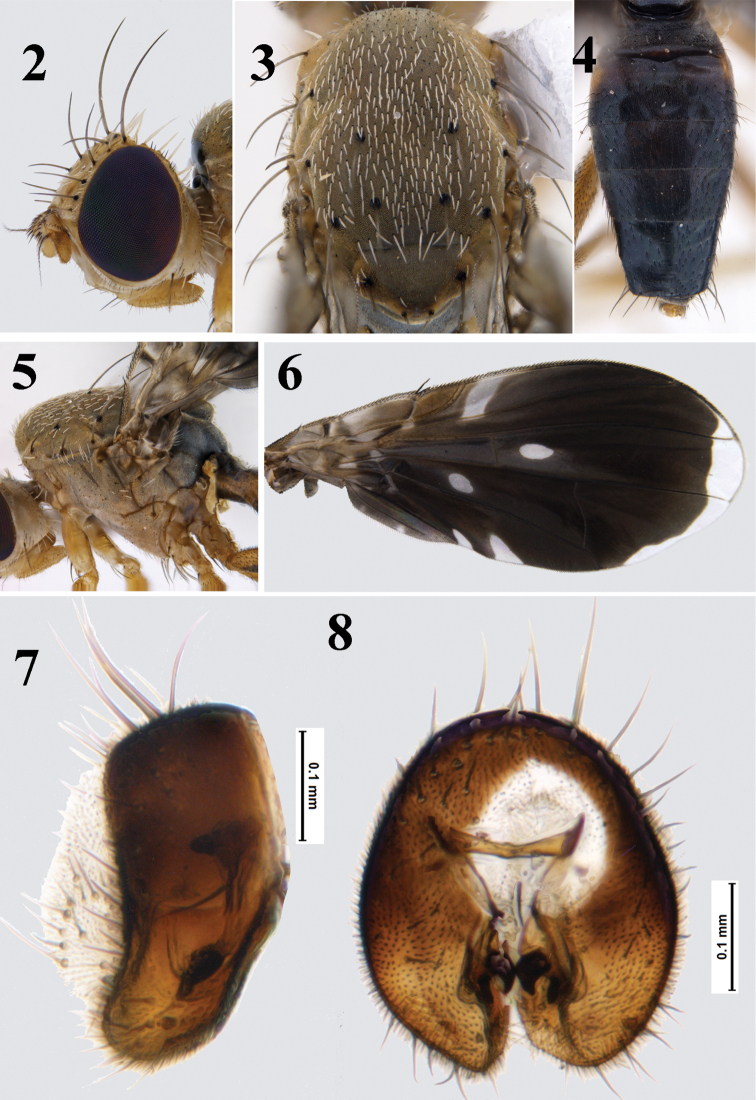
*Platensinarabbanii* David & Hancock, sp. nov. **2** head (lateral view) **3** thorax (dorsal view); **4** abdomen (dorsal view) **5** thorax (lateral view) **6** wing **7** epandrium (lateral view) **8** epandrium (posterior view).

***Head*** (Fig. 2): nearly as high as long; frons fulvous with three pairs of frontal setae and two pairs of orbital setae (posterior one white); ocellar triangle fuscous, with well developed ocellar setae; medial vertical seta black; lateral vertical seta, paravertical seta and postocellar seta lanceolate and white; postocular setae black interspersed with prominent, white lanceolate setae. Face fulvous without any markings. Scape and pedicel fulvous, first flagellomere less than half length of face, concolorous with frons, arista short pilose. Gena narrow, with prominent genal seta, subvibrissal setae present.

***Thorax*** (Figs 3, 5): Scutum uniformly grey microtrichose, with creamy-white setulae. Chaetotaxy well developed: 1 postpronotal, 1 presutural supra-alar, 1 anterior notopleural, 1 posterior notopleural, 1 postsutural supra-alar, 1 dorsocentral placed in line with postsutural supra-alar, 1 postalar, 1 intra-alar, and 1 prescutellar acrostichal seta. Scutellum brown with two pairs of scutellar setae; apical one less than half length of basal one. Anepisternum grey, with single seta near phragma; anepimeron grey, with single seta, katepisternum fulvous, with single seta; anatergite dark brown; katatergite and meron fulvous. Legs predominantly fulvous without any black/dark markings except basal, brown longitudinal streaks present on ventral surface of mid and hind femora; forefemur with single row of 4 ventral setae.

***Wing*** (Fig. 6) broad, with posterior margin evenly rounded, length 4.47 mm; length/width ratio-2.10, predominantly dark brown, with hyaline indentations and spots; cell bc hyaline, cell c predominantly moderate brown, with narrow basal and broader medial hyaline areas, pterostigma entirely moderate brown without any hyaline markings, cell r_1_ with one trapezoidal basal hyaline indentation not extending beyond vein R_2+3_, cell r_4+5_ with elongate subbasal hyaline spot, cell dm with large hyaline spot at basal third, cell cua with two short hyaline indentations not reaching vein CuA, and apical hyaline band extending from apex of cell r_2+3_ to cell m; anal lobe and alula dark brown.

***Abdomen*** (Fig. 4): Entirely black with yellowish orange patches laterally on tergites 1–2.

***Male genitalia*** (Figs 7, 8). Epandrium broad, lateral surstylus as broad as epandrium and connected at acute angle to epandrium (Fig. 7); apex of lateral surstylus blunt (in lateral view); proctiger short, smaller than epandrium; epandrium elongate oval in posterior view, medial surstylus with well developed prensisetae (lateral one broader than medial one).

**Female.** Unknown

##### Distribution.

Meghalaya (Northeast India).

##### Habitat.

Marshy grasslands.

##### Etymology.

The species is named after the collector, Rabbani Mehaboob K. It is a noun in apposition.

#### ﻿ Platensina flavistigma

Taxon classificationAnimaliaDipteraTephritidae

David & Hancock sp. nov.

C6AB1448-B895-59B5-9D5F-B7124012BA6C

http://zoobank.org/7512FEB5-3888-4CFF-8229-80C146706273

Platensina quadrula : Hancock 2012: 315 (misidentification, India record).

##### Type locality.

India: Karnataka, Bangalore, Attur.

##### Type data.

***Holotype*** male, pinned. Original label: “INDIA: Karnataka, Bangalore, Attur, 05.ii.2020, Sachin K (NIM)”. ***Paratypes*: India**: Periyakulam, 30.iii.2012, David, K. J. (1♂ NIM); India, Tamil Nadu, Thandikudi, C.R.S, 31.iii.2012, David, K. J. (1♂ NIM).

##### Diagnosis.

This species is similar to *P.quadrula* Hardy from Thailand, Cambodia and Vietnam in the presence of two broad quadrate areas in wing cell r_1_, an enlarged basal discal spot in cell r_4+5_ and large, broad hyaline indentations in cell cua. It can be differentiated by the lack of silvery facial spots and white rather than silvery parafacial stripes in males, as well as the angulate posterior wing margin, pterostigma predominantly fulvous/yellow and pale brown, and epandrium of uniform width throughout its length, whereas in *P.quadrula* the parafacial is silvery rather than white and facial silvery spots are present lateroventrally in males, the wing is evenly rounded posteriorly, the pterostigma is hyaline basally and dark brown apically, and the epandrium tapers apically. This species was mistakenly listed as *P.quadrula* from India by Hancock (2012).

##### Description.

**Figures 9–10.**
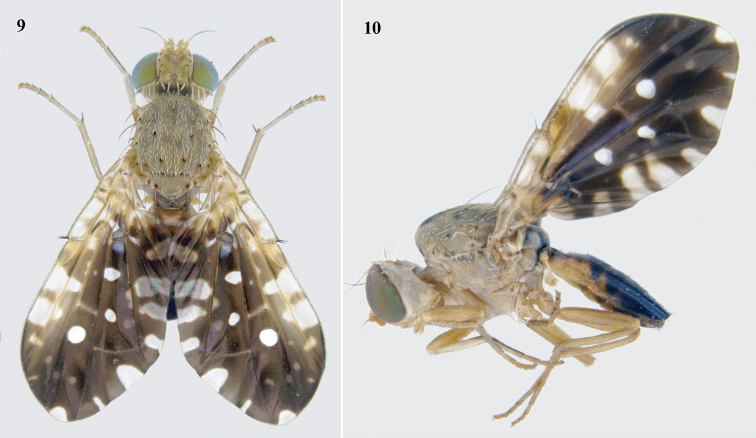
Habitus of *Platensinaflavistigma* David & Hancock, sp. nov. **9** dorsal view **10** lateral view.

**Male.** Medium-sized fly (4.10–4.99 mm long) with broad, angulate wing with fulvous markings (Figs 9, 10).

**Figures 11–16.**
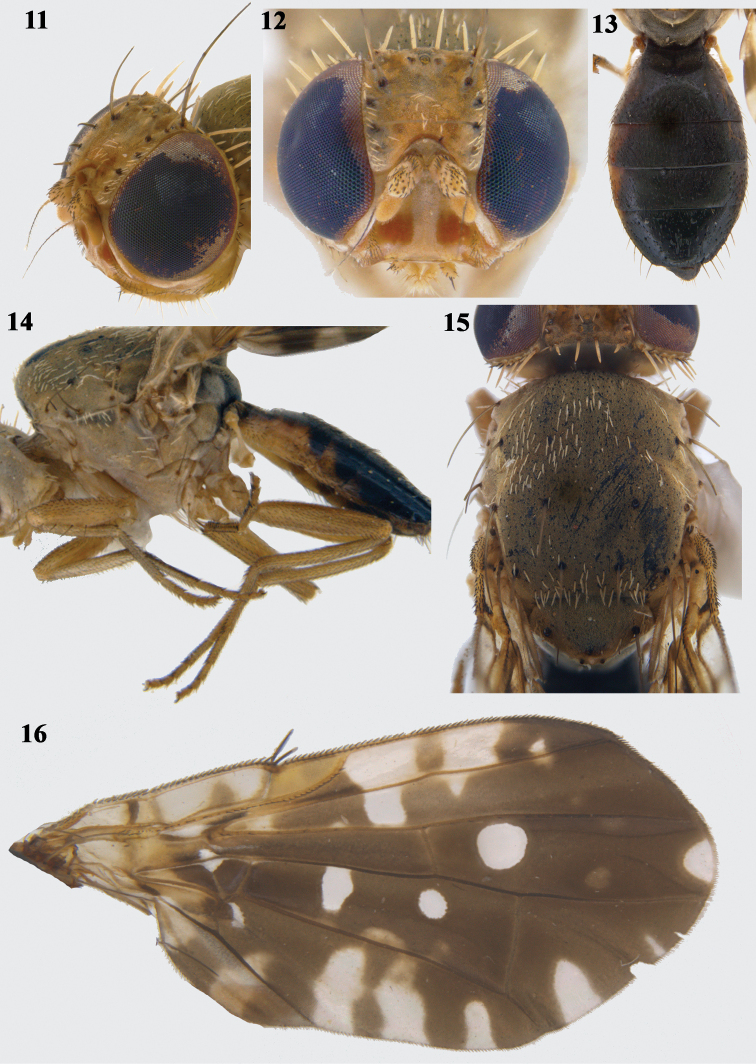
*Platensinaflavistigma* David & Hancock, sp. nov. **11** head (profile view) **12** head (frontal view) **13** abdomen (dorsal view) **14** thorax (lateral view) and legs **15** scutum **16** wing.

***Head*** (Figs 11, 12). as high as long; frons fulvous with three pairs of frontal setae and two pairs of orbital setae; ocellar triangle dark brown, with well-developed ocellar seta; medial vertical seta black; lateral vertical seta, paravertical seta and postocellar setae white; postocular setae black interspersed with prominent, white lanceolate setae. Face fulvous with broad orange-brown patches on ventral half of antennal groove. Scape and pedicel fulvous, first flagellomere shorter than face, concolorous with frons, arista short pilose. Parafacial alongside ventral half of face distinctly whitish. Gena narrow, with prominent genal seta, subvibrissal setae present.

***Thorax*** (Figs 14, 15). Scutum uniformly grey microtrichose with creamy-white setulae. Chaetotaxy well developed: 1 postpronotal, 1 presutural supra-alar, 1 anterior notopleural, 1 posterior notopleural, 1 postsutural supra-alar, 1 dorsocentral placed in line with postsutural supra-alar, 1 postalar, 1 intra-alar, and 1 prescutellar acrostichal seta. Scutellum grey with two pairs of scutellar setae; apical one less than half length of basal one. Anepisternum grey, with single seta near phragma; anepimeron grey, with single seta, katepisternum fulvous, with single seta; anatergite dark brown; katatergite and meron fulvous. Legs predominantly fulvous without any black/dark markings; forefemur with single row of 4 ventral setae.

***Wing*** (Fig. 16) broad, angulate, length 4.44–4.99 mm, length/width ratio, 1.92–2.05; predominantly dark brown with hyaline indentations and spots; cell bc hyaline, cell c predominantly hyaline with pale basal and medial infuscations, pterostigma fulvous basally, dark brown apically, cell r_1_ with two broad quadrate indentations and small posterior subapical hyaline spot, the proximal quadrate indentation extended to vein R_4+5_, cell r_4+5_ with large circular hyaline spot near base and large, semicircular hyaline apical spot, cell dm with two large hyaline spots, cell m with two marginal hyaline marks, subbasal one much larger than subapical one, cells r_2+3_ and r_4+5_ with indistinct and isolated pale brown subapical spots, cell cua with three large marginal hyaline indentations, basal two almost reaching vein CuA, and anal lobe with two broad subhyaline markings, proximal one crossing into cell cua.

***Abdomen*** (Fig. 13). Entirely black with yellowish orange patches laterally on tergites 1–3.

**Figures 17–19.**
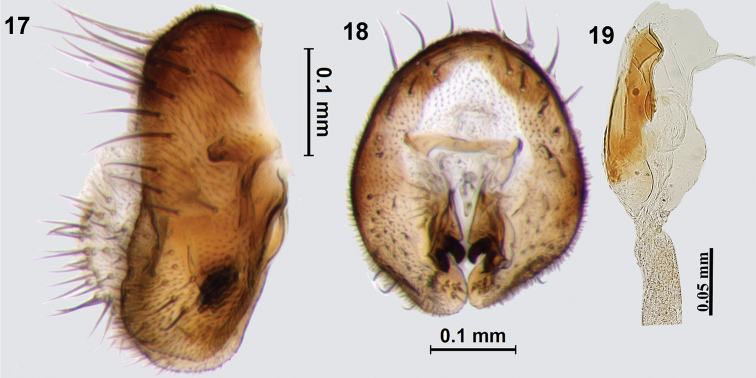
*Platensinaflavistigma* David & Hancock, sp. nov. **17** epandrium (lateral view) **18** epandrium (posterior view) **19** glans of phallus.

***Male genitalia*.** Epandrium broad, lateral surstylus as broad as epandrium (Fig. 17); apex of lateral surstylus blunt (in lateral view); proctiger short, smaller than epandrium; epandrium oval in posterior view (Fig. 18); medial surstylus shorter than lateral surstylus and with well developed prensisetae (lateral one broader than medial one). Phallus elongate (1.78 mm long), with sclerotised acrophallus (Fig. 19).

**Female.** Unknown

##### Etymology.

The specific name is derived from two Latin words *flavus* (=yellow) and *stigma* (=ptersotigma).

##### Distribution.

Karnataka and Tamil Nadu (southern India).

##### DNA Barcode.

NCBI GenBank accession number – MT019893 (1♂, India: Karnataka, Bangalore, Attur, 17.v.2018, Prabhu, G.)

### ﻿Taxonomic notes on other species of *Platensina* from India

#### ﻿ Platensina acrostacta

Taxon classificationAnimaliaDipteraTephritidae

(Wiedemann)

6D6AB33A-6606-5F68-A14E-1CDE148F00A8

Tephritis acrostacta Wiedemann, 1824: 54. Type locality: India-orientali [east India].
Ensina guttata Macquart, 1843: 387. Type locality Coromandel coast, Tamil Nadu, India.
Trypeta stellata Walker, 1849: 1030. Type locality North Bengal, India.
Trypeta voneda Walker, 1849: 1028. Type locality ‘Bahia, Brazil’ [*recte* Bengal, India].

##### Material examined.

India: 1♂, Karnataka, Tumkur, Kunigal, 05.iv.2013, Prabhu G. “leg”; 1♂, Karnataka, Bengaluru, Hebbal, 28.xi.2014, Prabhu G. “leg”; 1♂, Karnataka, Bengaluru, Attur, 24.iii.2016, Prabhu G. “leg”; India. 1♀, Karnataka, Chikkaballapur, 12.iv.2016, Prabhu G. “leg”; 1♂,1♀ Karnataka, Bengaluru, Attur, 16.v.2017, Prabhu G. “leg”; 1♂,1♀, Karnataka, Bengaluru, Attur, 20.vi.2017, Prabhu G. “leg”; 1♀, Karnataka, Bengaluru, Attur, 04.vii.2017, Prabhu G. “leg”; 1♂, Karnataka, Bengaluru, Attur, 07.ii.2018, Prabhu G. “leg”; 1♀, Karnataka, Bengaluru, Attur, 21.iii.2018, Prabhu G. “leg”; 1♂,1♀, Karnataka, Bengaluru, Attur, 24.iv.2018, Prabhu G. “leg”; 1♂, Karnataka, Bengaluru, Hebbal, 03.v.2018, Prabhu G. “leg”; 1♂,1♀, Karnataka, Bengaluru, Attur, 17.v.2018, Prabhu G. “leg”; 2♀, Karnataka, Bengaluru, Attur, 24.v.2018, Prabhu G. “leg”; 1♂, Karnataka, Bengaluru, Attur, 14.vi.2018, Prabhu G. “leg”; 1♂, Karnataka, Bengaluru, Attur, 14.x.2019, Sachin K. “leg”; 1♂, Kerala, Kasargod, C.P.C.R.I, 17.ii.2015, Prabhu, G.”leg”, 1♀,1♂, Tamil Nadu, Periyakulam, 30.iii.2012, David, K.J.”leg”, 1♂, Tamil Nadu, Thandikudi, C.R.S, 01.iv.2012, David, K.J. “leg”(NIM).

##### Diagnosis

**Figures 20–21.**
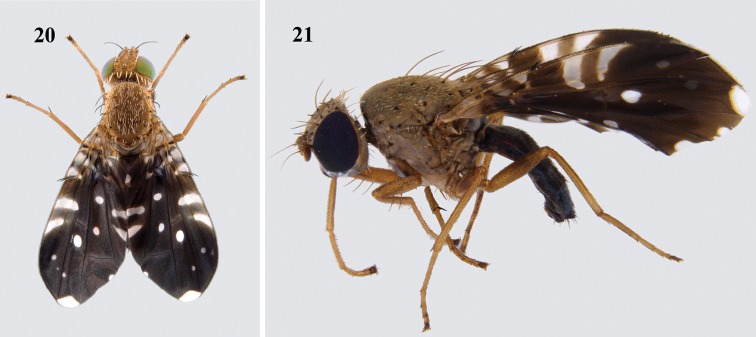
*Platensinaacrostacta* (Wiedemann) **20** habitus (dorsal) **21** habitus (lateral).

**Figures 22–30.**
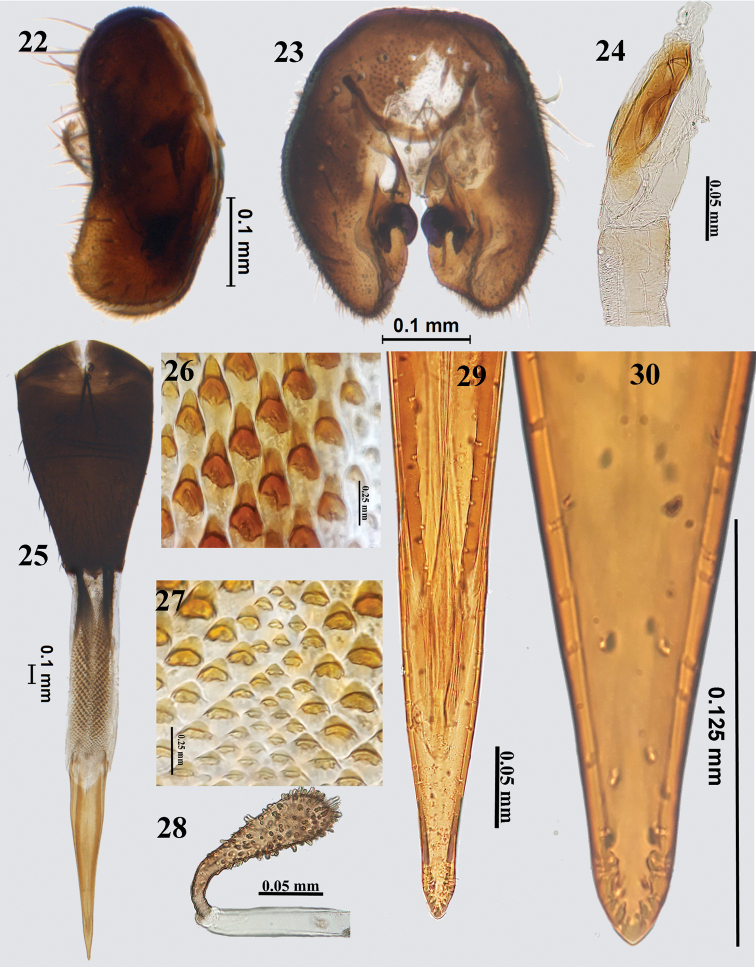
*Platensinaacrostacta* (Wiedemann) **22** epandrium (lateral) **23** epandrium (posterior) **24** glans of phallus **25** ovipositor **26** spicules on proximal end of eversible membrane **27** spicules on distal end of eversible membrane **28** spermatheca **29** aculeus **30** aculeus tip (100×).

**(Figs 20–30).** A medium-sized species separated from the similar *P.fulvifacies* Hering largely by the black face in males; length of male (4.29–4.47 mm), of female (5.45–5.49 mm). This species was adequately described by Hardy (1973) except for detailed structures of male and female postabdomen. Epandrium broad, not demarcated from surstylus (profile view); proctiger smaller than epandrium. Epandrium and surstyli elongate oval in posterior view; prensisetae well developed. Phallus elongate (2.71 mm); glans with a sclerotised rod. Oviscape (1.21 mm) dark brown to black, dorsoventrally flattened; eversible membrane shorter than oviscape (0.96 mm) with conical spicules in the proximal region and triangular spicules towards distal end. Aculeus (1.01 mm) as long as eversible membrane and with triangulate apex. Spermatheca elongate oval with numerous papillae.

##### Distribution.

This species is known from India (Karnataka, Tamil Nadu and Kerala) and Sri Lanka to Cambodia (Hancock 2012).

##### DNA Barcode.

NCBI GenBank accession number – MT019891 (1♂, India: Karnataka, Bangalore, Attur, 29.v.2019, Sachin, K.).

#### ﻿ Platensina fulvifacies

Taxon classificationAnimaliaDipteraTephritidae

Hering

49D86386-123D-57D7-A417-73943FC0E09E

Platensina fulvifacies Hering, 1941: 71. Type locality Lonaula, Maharashtra, India.

##### Diagnosis.

This species is similar to *P.acrostacta* but can be differentiated primarily by the yellow face in males and larger basal spot in cell dm. Specimens were not available for study but photographs of both sexes have been examined: 2♂, 2♀, India: Rajasthan, Jodhpur District, 10 km SW Jodhpur, Machia Safari Pk, Malaise in dry wash 29.II–5.III.2008, 300 m, 26°18.60'N, 72°58.71'E (in California Academy of Sciences, San Francisco, California, USA).

##### Distribution.

This endemic Indian species is known only from Maharashtra and Rajasthan.

#### ﻿ Platensina platyptera

Taxon classificationAnimaliaDipteraTephritidae

Hendel

13C1560B-0286-59E1-A532-CE0324B851AC

Platensina platyptera Hendel, 1915: 461. Type locality Taihorin, Taiwan.
Platensina malaita Curran, 1936: 29. Type locality Tai Lagoon, Malaita, Solomon Is.
Platensina dubia Malloch, 1939: 459. Type locality Gordonvale, Qld, Australia.
Platensina amplipennis : authors, *nec* Walker, 1860. Misidentifications.

##### Material examined.

India: 1♀, Karnataka, Bengaluru, G.K.V.K, 02.ii.2012, David K.J. “leg”; 1♂, A&N Islands, Middle Andamans, Kadamtala, 09.iii.2012, David, K.J. “leg”; 1♀, Karnataka, Mandya, Maddur, 09.i.2013, David, K.J. “leg”; 1♀, Karnataka, Uttara Kannada, Dandeli, 15.i.2015, Rajesh S. “leg”; 1♀, Meghalaya, Mawlynnong Road, 12.x.2019, David, K.J. “leg” (NIM).

##### Diagnosis

**Figures 31–32.**
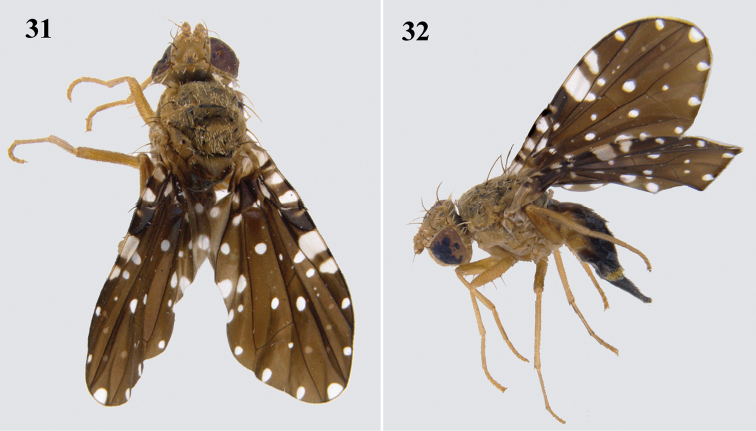
*Platensinaplatyptera* Hendel **31** habitus (dorsal) **32** habitus (lateral).

**(Figs 31, 32).** This species was originally described by Hendel (1915) from Taiwan. It was recorded from India by Hancock (2012), based on photographs by the senior author. It is a medium-sized species with broad quadrate hyaline markings in cells c and r_1_, round hyaline spots along the wing margin and in almost all the cells. It is similar to *P.zodiacalis* (Bezzi) but can be differentiated by the presence of apical scutellar setae.

##### Male genitalia.

**Figures 33–41.**
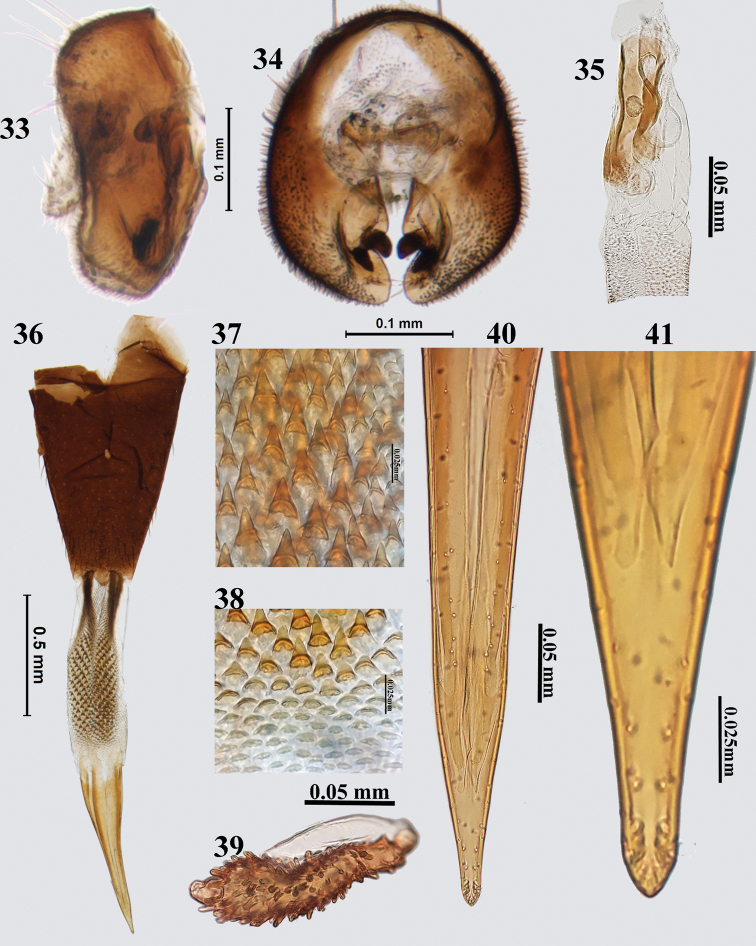
*Platensinaplatyptera* Hendel **33** epandrium (lateral) **34** epandrium (posterior view) **35** glans of phallus **36** ovipositor **37** spicules on proximal end of eversible membrane **38** spicules on distal end of eversible membrane **39** spermatheca **40** aculeus **41** aculeus tip (100×).

Epandrium (Fig. 33) dark brown, heavily sclerotised, with no demarcation from lateral surstylus (in profile view); epandrium and surstyli oval in posterior view, with well developed prensisetae (Fig. 34). Phallus elongate (1.54 mm long including glans); glans stout, with broad sclerotised rod (Fig. 35).

##### Female genitalia.

Oviscape 0.99 mm long, dark brown, conical, dorsoventrally flattened (Fig. 36); eversible membrane 0.83 mm long, shorter than oviscape, with conical spicules along entire length except for a few rows of flattened ones distally (Figs 37, 38); taeniae short, dark brown, 0.25 of length of eversible membrane. Aculeus shorter than eversible membrane, not dorsoventrally flattened, curved at its proximal end; apex of aculeus pointed and conical (Figs 40, 41). Spermatheca brown, club-shaped, with numerous papillae (Fig. 39).

##### Distribution.

This species is widespread from India (Kerala, Karnataka, Meghalaya, Andaman and Nicobar Islands) to Japan and Australasia (Hancock 2012).

##### DNA Barcode.

NCBI GenBank accession number – MW448367 (1♂, India: Kerala, Kannur, Aaralam, 13.i.2020, David, K. J.).

#### ﻿ Platensina tetrica

Taxon classificationAnimaliaDipteraTephritidae

Hering

45293323-B7B5-512F-A945-8C6968C9C60D

Platensina tetrica Hering, 1939a: 179. Type locality Trichinopolis, Tamil Nadu, India.
Platensina fukienica Hering, 1939b: 146. Type locality Fujian, China.

##### Diagnosis.

This species was adequately described by Hering (1939a, b) and is characterised by the reduced and often indistinct hyaline markings. Indian specimens were not available for study but wings of both *P.tetrica* Hering and *P.fukienica* Hering, considered synonyms by Hancock (2012), were illustrated by Wang (1998).

##### Distribution.

This species is known in India only from the type locality in Tamil Nadu. Elsewhere, it is known from China, Taiwan, Vietnam and West Malaysia (Hancock 2012).

#### ﻿ Platensina zodiacalis

Taxon classificationAnimaliaDipteraTephritidae

(Bezzi)

60D96A31-7909-5F43-A4A4-39751C22FC9D

Tephritis zodiacalis Bezzi, 1913: 163. Type locality Calcutta [Kolkata], India.
Platensina zodiacalis : Hering 1956: 69. Lapsus calami.
Platensina zodiacalis : Hendel 1915: 461.

##### Material examined.

India: 1♂, Karnataka, Madikeri, Chettalli, 05.xi.2012, David K.J. “leg”; 1♀, Assam, Dibrugarh, 07.xi.2014, Ramesh Kumar A. “leg”; 1♂, Karnataka, Kidu, CPCRI, 19.ii. 2015, David K.J. “leg”; 1♂, Karnataka, Chettalli, C.H.E.S, 03.ii.2021, David K.J. “leg”; 1♂, Assam, Barpeta, K.V.K, 09.iii.2021, Sachin K. “leg”; 1♂, Assam, Barpeta, K.V.K, 10.iii.2021, David. K.J. “leg”; 1♀, Assam, Chirang, K.V.K, 13.iii.2021, Sachin K. “leg”; 1♂, Assam, Golpara, 14.iii.2021, Rabbani M.K. “leg” (NIM).

##### Diagnosis.

**Figures 42–43.**
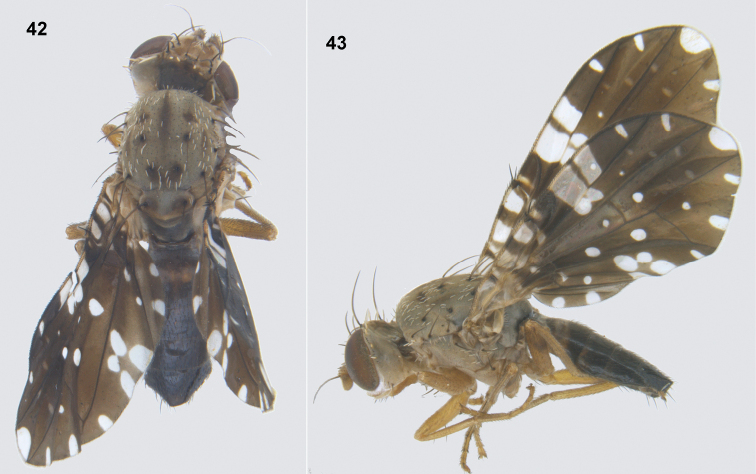
*Platensinazodiacalis* (Bezzi) **42** habitus (dorsal) **43** habitus (lateral).

This species was adequately described by Bezzi (1913) except for the postabdominal structures. It is almost inseparable from *P.platyptera* except for the presence of only basal scutellar setae instead of both apical and basal setae as in *P.platyptera* (Figs 42, 43).

##### Male genitalia.

**Figures 44–51.**
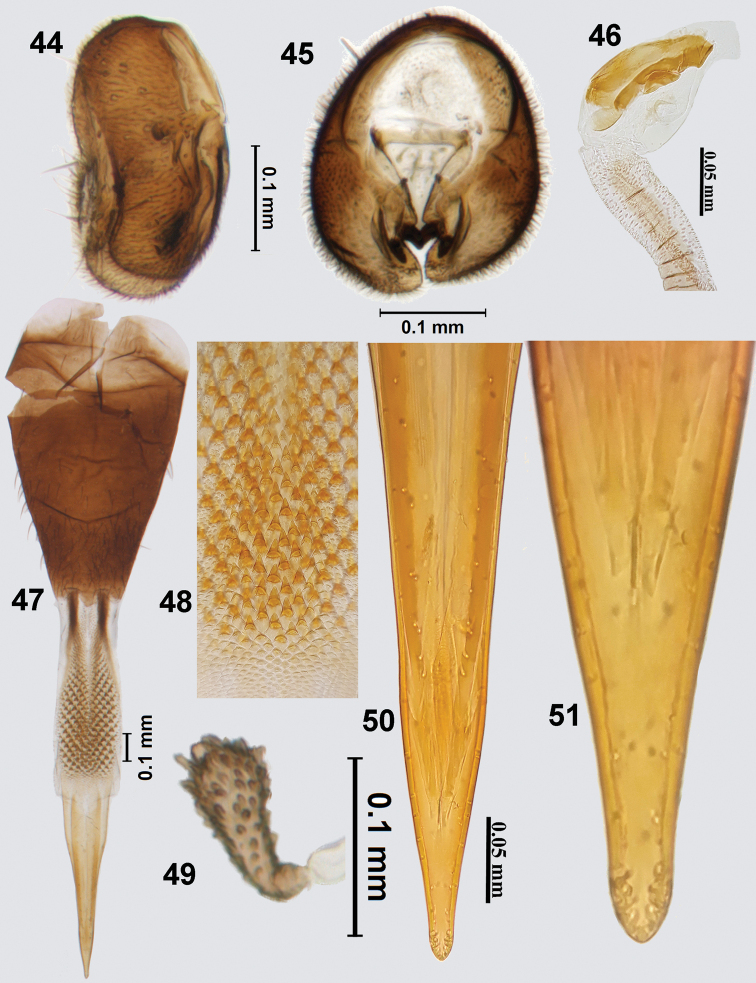
*Platensinazodiacalis* (Bezzi) **44** epandrium (lateral) **45** epandrium (posterior view) **46** glans of phallus **47** ovipositor **48** spicules on eversible membrane **49** spermatheca **50** aculeus **51** aculeus tip (100×).

Epandrium (Fig. 44) brown, sclerotised, with no demarcation from lateral surstylus (in profile view); epandrium and surstyli oval in posterior view, with well-developed prensisetae (Fig. 45). Phallus 1.39 mm long including glans; glans stout, with broad sclerotised rod (Fig. 46).

##### Female genitalia.

Oviscape 0.92 mm long, dark brown, conical, dorsoventrally flattened (Fig. 47); eversible membrane 0.72 mm long, shorter than oviscape, with conical spicules along entire length except for a few rows of flattened ones distally (Fig. 48); taeniae short, dark brown, 0.25 length of eversible membrane. Aculeus shorter than eversible membrane, 0.63 mm long, dorsoventrally flattened; apex of aculeus rounded (Figs 50, 51). Spermatheca brown, club-shaped, with numerous papillae (Fig. 49).

##### Distribution.

This species is widespread from India (Karnataka, Assam) and Sri Lanka to southern China and Australia (Hancock 2012).

### ﻿Evolutionary divergence among the Indian species of *Platensina*

Table 1 shows the pair-wise evolutionary divergence/distance between four sequences available in NCBI database for three species namely *P.acrostacta*, *P.platyptera* and *P.flavistigma*. Evolutionary distance between *P.flavistigma* David & Hancock, sp. nov. and *P.acrostacta* is 0.0429 and between *P.flavistigma* and *P.platyptera* is 0.0759 which reveals that *P.flavistigma* is a distinct species in *Platensina* based on the available sequences. Among the species included in the analysis, evolutionary distance between *P.platyptera* and *P.acrostacta* was the highest (0.0837), which is evident in the morphological differences between these two species with respect to facial markings and wing pattern.

**Table 1.** Evolutionary divergence among the Indian species of *Platensina*.

Species name with accession numbers	*P.acrostacta* (MH748566)	*P.platyptera* (MW448367)	*P.flavistigma* (MT019893)	*P.acrostacta* (MT019891)
*P.acrostacta* (MH748566)				
*P.platyptera* (MW448367)	0.0837			
*P.flavistigma* (MT019893)	0.0429	0.0759		
*P.acrostacta* (MT019891)	0.0036	0.0882	0.0470	0.0000

## Supplementary Material

XML Treatment for Platensina

XML Treatment for Platensina rabbanii

XML Treatment for Platensina flavistigma

XML Treatment for Platensina acrostacta

XML Treatment for Platensina fulvifacies

XML Treatment for Platensina platyptera

XML Treatment for Platensina tetrica

XML Treatment for Platensina zodiacalis
